# Complete Genome Sequence of Cyanobacterium *Geminocystis* sp. Strain NIES-3708, Which Performs Type II Complementary Chromatic Acclimation

**DOI:** 10.1128/genomeA.00357-15

**Published:** 2015-05-07

**Authors:** Yuu Hirose, Mitsunori Katayama, Yoshiyuki Ohtsubo, Naomi Misawa, Erica Iioka, Wataru Suda, Kenshiro Oshima, Mitsumasa Hanaoka, Kan Tanaka, Toshihiko Eki, Masahiko Ikeuchi, Yo Kikuchi, Makoto Ishida, Masahira Hattori

**Affiliations:** a Department of Environmental and Life Sciences, Toyohashi University of Technology, Tempaku, Toyohashi, Aichi, Japan; b Electronics-Inspired Interdisciplinary Research Institute (EIIRIS), Toyohashi University of Technology, Tempaku, Toyohashi, Aichi, Japan; c College of Industrial Technology, Nihon University, Narashino, Chiba, Japan; d Department of Environmental Life Sciences, Graduate School of Life Sciences, Tohoku University, Katahira, Sendai, Miyagi, Japan; e Center for Omics and Bioinformatics, Graduate School of Frontier Sciences, The University of Tokyo, Kashiwa, Chiba, Japan; f Division of Applied Biological Chemistry, Graduate School of Horticulture, Chiba University, Matsudo, Chiba, Japan; g Chemical Resources Laboratory, Tokyo Institute of Technology, Midori-ku, Yokohama, Kanagawa, Japan; h Department of Life Sciences (Biology), The University of Tokyo, Meguro, Tokyo, Japan; i Graduate School of Advanced Science and Engineering, Waseda University, Shinjuku-ku, Tokyo, Japan

## Abstract

To explore the variation of the light-regulated genes during complementary chromatic acclimation (CCA), we determined the complete genome sequence of the cyanobacterium *Geminocystis* sp. strain NIES-3708. Within the light-regulated operon for CCA, we found genes for phycoerythrin but not phycocyanin, suggesting that this cyanobacterium modulates phycoerythrin composition only (type II CCA).

## GENOME ANNOUNCEMENT

Certain cyanobacterial species modulate the composition of their light-harvesting antennae, phycoerythrin and phycocyanin, in response to green and red light, a phenomenon termed complementary chromatic acclimation (CCA) (1, 2). Recent studies have shown that a cyanbacteriochrome-type photoreceptor (3, 4), CcaS or RcaE, perceives green and red light and regulates the expression of these antenna genes (5–8). Interestingly, the antenna gene sets that are regulated by CcaS or RcaE are different among the analyzed cyanobacterial species *Synechocystis* sp. PCC 6803, *Nostoc punctiforme* ATCC 29133, and *Fremyella diplosiphon*.

To explore the variation in the light-regulated genes during CCA, we performed whole-genome sequencing of another CCA-capable cyanobacterium, *Geminocystis* sp. strain NIES-3708, which was isolated from a Japanese freshwater stream. We used the GS FLX+ (Roche) and MiSeq (Illumina) systems for sequencing. For GS FLX+, a shotgun library and an 8-kb paired-end library were prepared using a GS FLX+ library preparation kit (Roche) and GS FLX paired-end kit (Roche), respectively. The libraries were sequenced on the GS FLX+ instrument, yielding 309,976 shotgun reads and 222,244 paired-end reads. For MiSeq, an 800-bp paired-end library and an 8-kbp mate-pair library were prepared using the TruSeq DNA PCR-free sample preparation kit (Illumina) and Nextera mate-pair sample preparation kit (Illumina), respectively. The libraries were sequenced on the MiSeq instrument with the MiSeq reagent kit version 2 (500 cycles; Illumina), which yielded 668,946 paired-end reads and 539,927 mate-pair reads. The MiSeq reads were filtered using ShortReadManager (9), based on a 17-mer frequency. The FLX+ and MiSeq reads were then assembled using Newbler version 2.8 (Roche). The sequence gaps between the contigs were determined *in silico* using GenoFinisher and AceFileViewer (9).

The complete genome sequence of *Geminocystis* sp. NIES-3708 comprises one chromosome of 3,883,409 bp and five plasmids of 60,790, 57,874, 17,905, 16,994, and 5,106 bp. The G+C content of the genome was calculated to be 32.3%. A total of 3,641 protein-coding genes, 6 rRNA genes, and 45 tRNA genes were predicted using Rapid Annotations using Subsystems Technology (RAST) (10). We identified the genes of cyanobacteriochrome CcaS and cognate transcriptional regulator CcaR for CCA (5, 6). The light-regulated gene operon is composed of the linker (CpeC) and the regulator (CpeR) of phycoerythrin but not the hydrophobic linker of phycocyanin (CpcL) (11). This suggests that *Geminocystis* sp. NIES-3708 modulates phycoerythrin content only, which classifies it as an organism performing type II CCA (12). These data will bring insights into the molecular basis and evolution of CCA among cyanobacteria.

### Nucleotide sequence accession numbers. 

The complete genome sequence of *Geminocystis* sp. NIES-3708 has been deposited in the DNA Data Bank of Japan under the accession numbers AP014815 through AP014820.
